# Integrated transcriptome and metabolome analyses reveal key genes regulating jujuboside biosynthesis in *Ziziphus jujuba* var. *spinosa*

**DOI:** 10.3389/fpls.2025.1708851

**Published:** 2026-01-08

**Authors:** Dali Geng, Xiaozhou Yang, Xinhong Wang, Xiaojun Chi, Xiaohan Tang, Xiaojun Ma, Xuexiang Li, Menglin Pu, Jing Shu

**Affiliations:** Department of Forestry Engineering, Shandong Agriculture and Engineering University, Zibo, China

**Keywords:** *Ziziphus jujuba* var. *spinosa*, jujuboside biosynthesis, triterpenoid biosynthesis, transcriptome and metabolic analyses, CYP450, UDP- glucuronosyltransferase

## Abstract

The seeds of *Ziziphus jujuba* var. sp*inosa* are a traditional Chinese medicine for treating insomnia, with jujuboside A and jujuboside B as their core bioactive components. However, the biosynthetic pathways and regulatory mechanisms of these triterpenoid saponins remain poorly understood. In this study, we investigated the accumulation patterns of jujube saponins across 10 varieties at 45, 65, and 85 days after flowering (DAF). Integrated transcriptomic and metabolomic analyses revealed 2,040 differentially expressed genes (DEGs) and 1,593 differentially accumulated metabolites (DAMs). Among these, 35 core DEGs were consistently significant across comparisons, enriched in saponin metabolism, monooxygenase activity, and glycosylation processes. A Uridine Diphosphate (UDP)-glucosyltransferase and two CYP450 family members exhibited expression patterns positively correlated with saponin accumulation, showing upregulation during 45 and 65 DAF and suppression by 85 DAF. qRT-PCR validation confirmed their higher expression in high-saponin varieties. Additionally, downregulated stress–response genes in high-saponin varieties suggested a potential link between abiotic stress tolerance and enhanced saponin production. These findings identify key glycosyltransferase and cytochrome P450 genes potentially governing jujuboside biosynthesis, providing critical insights for metabolic engineering and quality improvement of *Z. jujuba*.

## Introduction

The number of individuals with insomnia disorders in China is rapidly increasing. This growth in the population suffering from sleep disorders has led to a soaring market demand for sedative drugs (Zhao et al., 2020). Seeds of *Ziziphus jujuba* var. sp*inosa* (*Z. jujuba*), a traditional Chinese medicinal herb used for treating insomnia, have consequently experienced rapidly increasing demand and price (Hua et al., 2021). However, despite the strong demand for *Z. jujuba*, its production has long faced challenges. At the germplasm level, the production of *Z. jujuba* has historically relied on the collection of wild resources, lacking systematic genetic improvement and standardized cultivation techniques. Additionally, long-term neglect of this traditional Chinese medicine means that research on *Z. jujuba* breeding and the mechanisms underlying its medicinal efficacy remains insufficient. The biosynthetic pathways of its core active components, jujuboside A and jujuboside B, remain unclear, and key enzyme genes and regulatory networks within the related secondary metabolic pathways have yet to be elucidated. This quality uncertainty severely hinders the development of standardized formulations and the stability of pharmaceutical and health product efficacy, thereby impeding the promotion and popularization of *Z. jujuba*-based sedative drugs and health products and presenting a significant challenge for the healthy development of the *Z. jujuba* industry.

The key to overcoming these challenges lies in deciphering the molecular basis of *Z. jujuba’s* medicinal efficacy formation. Unraveling the biosynthetic mechanisms of *Z. jujuba’s* active components requires focusing on the discovery of its key rate-limiting enzyme systems. Although the aglycone structure of jujuboside A/B shares homology with dammarane-type triterpenes, the formation mechanism of its characteristic ketal ring structure is specific (Li et al., 2023). Current research indicates that this molecule undergoes oxygenation at the C-16 and C-30 positions of dammarenediol-II, followed by aldol condensation and ketal cyclization to form the C16–C30 and C16–C23 rings. This series of oxidative modification reactions is highly dependent on hydroxylase catalysis. Notably, in *Panax ginseng*, the CYP450 superfamily (particularly members of the CYP716A subfamily) has been confirmed to precisely regulate the hydroxylation sites and reaction sequence of dammarane-type triterpenes. Its functional diversity provides an important reference for deciphering the jujuboside biosynthetic network (Han et al., 2011, 2012, 2013) and has been successfully applied in the artificial cellular synthesis of ginsenosides, achieving the production of intermediates (Zhu et al., 2014; Zhao et al., 2016; Song et al., 2020), aglycones (Li D. et al., 2019), or saponins (Wei et al., 2015; Wang et al., 2015; Zhuang et al., 2017) in microbial cells such as *Escherichia coli*, *Bacillus subtilis*, *Pichia pastoris*, *Yarrowia lipolytica*, and *Saccharomyces cerevisiae*. However, despite also being dammarane-type tetracyclic triterpenoids, the functional divergence of CYP450 homologs corresponding to those in ginseng and their catalytic specificity for the characteristic ketal structure in jujuboside biosynthesis remain unknown. The absence of these key enzymes directly hinders the complete mapping of the molecular regulatory network governing the jujuboside biosynthetic pathway.

Biosynthesis of dammarane-type triterpenoids is related to Uridine Diphosphate (UDP)-glucosyltransferase and CYP450 (Shin and Oh, 2016). CYP450 enzymes perform complex modifications such as hydroxylation and oxidation on the carbon ring framework of triterpenoids and are key enzymes in the dammarane-type triterpenoid biosynthesis pathway. In *P. ginseng*, 11 CYP450 genes related to ginsenoside biosynthesis were discovered and cloned by Zeng et al. (2018). Among these genes, CYP716A47 can increase expression in response to methyl jasmonate induction. Furthermore, when transgenic ginseng plants with an overexpressed SS gene are produced, the yield of saponins in the roots increases. By introducing CYP716A47 into brewing yeast, the expressed recombinant protein can catalyze the C-12 hydroxylation of dammarane-2,4-diol and convert it into protospondiols. When DS and CYP716A47 are simultaneously introduced into brewing yeast, protospondiol production was detected in the recombinant strain (Han et al., 2011).

In *Panax notoginseng*, 15 CYP450 genes were discovered by Luo et al. (2011). Among these genes, Pn00158 is a homolog of CYP716A47 in *P. ginseng* (Li D. et al., 2019). UDP-glucosyltransferase catalyzes the glycosylation reaction, the final step in jujube saponin biosynthesis. This process involves transferring sugar groups to the aglycone of the saponin to form glycosidic bonds. In *P. ginseng*, UDP-glucuronosyltransferase Pg1 (UGTPg1) catalyzes the biosynthesis of ginsenoside C-K (Yan et al., 2014); UGTPg45 and UDP-glucuronosyltransferase Pg29 (UGTPg29) catalyze the biosynthesis of ginsenoside Rh2 and Rh3 (Wang et al., 2015); and UGTPg1 and UGTPg100 catalyze the biosynthesis of ginsenoside Rh1 and F1 (Wei et al., 2015).

In this paper, we investigated the accumulation patterns of jujube saponins across 10 *Z. jujuba* varieties at 45, 65, and 85 days after flowering (DAF). We performed integrated transcriptomic and metabolomic analyses on varieties exhibiting contrasting saponin profiles. Differential expression analysis identified 2,040 differentially expressed genes (DEGs) and 1,593 differentially accumulated metabolites (DAMs), with 35 core DEGs consistently significant across comparisons. Gene Ontology (GO) enrichment revealed that these DEGs were associated with saponin metabolism, monooxygenase activity, and glycosylation processes. Co-expression analysis highlighted three candidate genes whose temporal expression patterns correlated with saponin accumulation. These genes were upregulated during the active saponin biosynthesis stages (45–65 DAF) but suppressed by 85 DAF. Their involvement in jujuboside biosynthesis was further supported by quantitative reverse transcription polymerase chain reaction (qRT-PCR) validation and by observed correlations with precursor metabolite levels. Finally, the potential linkage between abiotic stress tolerance and enhanced saponin production was indicated by downregulated stress–response genes in high-saponin varieties.

## Methods

### Plant materials

*Z. jujuba* var. *spinosa* were planted in Guangrao County, Dongying City, Shandong Province, China (118.45°E, 37.08°N) with 1.2‰ soil salinity. We selected 5-year-old plants for seed collection. Labels indicating the date were hung on buds when they were about to bloom, and fruits were then harvested and frozen in liquid nitrogen at 35, 45, 55, 65, 75, and 85 DAF. Epicarp, sarcocarp, and endocarp were removed with a nutcracker in liquid nitrogen to maintain frozen conditions. Seeds were collected and stored at − 80°C. Ten varieties of the hybrid from the crossbreeding of “Madu” with the “Pufeng” variety were selected; for each variety, five individuals were chosen, and 10 seeds were harvested from each individual.

### Jujube saponin content measurement

Seeds were crushed in a mixer mill (MM 400, Retsch) containing small steel balls in liquid nitrogen for 1.5 min at 30 Hz. The powder was divided into equal parts. One part was stored at − 80°C, and the other part was freeze-dried in a vacuum freeze dryer (LGJ-18D, The Fourth Ring, Beijing, China). The lyophilized powder was stored at − 80°C. Three seeds from different individuals were used as biological replicates.

Jujuboside A, jujuboside B, and jujubogenin were purchased from Desite Biotech (Chengdu, Sichuan Province, China). Lyophilized powder was used for the High Performance Liquid Chromatography with Evaporative Light Scattering Detector (HPLC-ELSD) analysis. A 100-mg sample was extracted with 1.5 mL of 50% methanol in a sonicator bath for 1 h and centrifuged at 12,000 × *g* for 5 min to remove debris. The supernatant was filtered through a 0.2-μm filter before injection. High Performance Liquid Chromatography with Evaporative Light Scattering Detector (HPLC-ELSD) was performed with the Vanauish Core Duo (Thermo Fisher, Waltham, MA, USA). Separation was achieved with a 150 × 2.1 mm, 3 µm C18–120 column (Shimadzu, Tokyo, Japan) using the following gradient: 0.2% acetic acid in water (32%) vs. methanol (68%) run at 1 mL min^−1^ and a column temperature of 30°C. The ELSD detection shows an atomization temperature of 45°C and a gas flow rate of 1.6 L min^−1^. Each group had three biological repeats.

### Transcriptome and metabolome analyses

Seeds of 45 and 65 lyophilized powder were used for metabolome and transcriptome analyses. The metabolome analysis was performed by Metware Biotechnology (Wuhan, Hubei Province, China) using methods described by (Chen et al., 2018). Metabolite profiling was conducted using an ExionLC™ AD UPLC system coupled to a QTRAP^®^ 6500+ mass spectrometer (SCIEX, Danaher Corporation, USA). Chromatographic separation was performed on an Agilent SB-C18 column (1.8 µm, 2.1 × 100 mm) with a mobile phase comprising 0.1% formic acid in water (solvent A) and 0.1% formic acid in acetonitrile (solvent B). The gradient program initiated at 95% A/5% B, linearly transitioned to 5% A/95% B over 9 min, held at this composition for 1 min, then re-equilibrated to 95% A/5% B within 1.1 min, followed by a 2.9-min stabilization period. Separation was performed at a flow rate of 0.35 mL min^−1^, column temperature of 40°C, and injection volume of 2 µL. Electrospray ionization parameters included a source temperature of 500°C, a spray voltage of ± 5,500 V (positive/negative mode), and gas pressures of 50 psi (GS1), 60 psi (GS2), and 25 psi (CUR). Mass spectrometry utilized high CAD energy with medium-pressure nitrogen collision gas. Data acquisition occurred in time-scheduled MRM mode, where declustering potentials and collision energies were optimized for each metabolite transition and monitored according to the corresponding elution windows. Transcriptome sequencing analysis was performed by Metware Biotechnology (Wuhan, China) following the protocol described by Chen et al. (2018). Total RNA from plant tissues was extracted using the CTAB-PBIOZOL method combined with ethanol precipitation, while RNA from animal tissues was processed using Trizol reagent. Following extraction, RNA pellets were dissolved in 50 μL of DEPC-treated nuclease-free water. RNA concentration was precisely quantified using a Qubit fluorometer, and RNA integrity was assessed using a Qsep400 biofragment analyzer to ensure samples met library construction requirements.

For mRNA library preparation, polyadenylated mRNAs were first enriched using Oligo(dT) magnetic beads based on the polyA tail structure characteristic of eukaryotic mRNAs. The purified mRNAs were then fragmented into appropriate lengths under controlled temperature conditions using a fragmentation buffer. First-strand complementary DNA (cDNA) synthesis was performed via reverse transcription with random hexamer primers, followed by second-strand cDNA synthesis, during which dUTP was incorporated instead of dTTP during end repair and dA-tailing. This strategic substitution enables strand-specific sequencing, as subsequent PCR amplification selectively degrades uracil-containing strands. Sequencing adapters were ligated to the double-stranded cDNA fragments, followed by purification and size selection using SPRI beads to obtain libraries with 250–350 bp inserts. The adapter-ligated products underwent PCR amplification and final purification, with elution performed in nuclease-free water.

After library construction, concentration was quantified using Qubit, and fragment size distribution was validated using the Qsep400 analyzer. Qualified libraries were pooled according to effective concentration and target sequencing output, then subjected to paired-end 150 bp (PE150) sequencing on an Illumina platform. The sequencing-by-synthesis principle involves bridge amplification of DNA fragments on the flow cell to generate clusters. During each cycle, fluorescently labeled dNTPs and DNA polymerase are introduced, with nucleotide incorporation releasing characteristic optical signals. The sequencer captures these signals and converts them into base calls through dedicated software, generating complete sequence reads for the target fragments. Raw data were subjected to adapter trimming and low-quality read filtration prior to downstream gene expression analysis.

Samples at 45 and 65 DAF were used for metabolome and transcriptome analyses. Each group had three biological repeats, with nine seeds from three different individuals per replicate.

### qRT-PCR analysis, gene cloning, and phylogenetic analysis

The nonlyophilized powder of all samples was used for the qRT-PCR analysis. A 500-mg portion of this powder was used to extract RNA with the Polysaccharides and Polyphenolics-rich RNAprep Pure Plant Kit (Cat. No. DP441, Tiangen Biotech, Beijing, China). A total of 2 µg of RNA was used to synthesize the first-strand cDNA with the PrimeScript™ First Strand cDNA Synthesis Kit (Takara, Shiga, Japan). The cDNA reaction mixture was diluted five times, and 5 µL was used in the 20-µL PCR reaction. PCR reactions included a preincubation step at 95°C for 2 min, followed by 45 cycles of denaturation at 95°C for 15 s, annealing at 54°C for 30 s, and extension at 72°C for 30 s. All reactions were performed in the QuantStudio™ 5 Food Safety Real-Time PCR System using TB Green Fast qPCR Mix (Takara) with ROX reference dye. Each experiment had nine replicates (three technical replicates for each biological replicate). The relative expression levels were calculated using the 2^−ΔΔCt^ method.

The cDNA reaction mixture was used for cloning. Gene cloning was performed with TaKaRa Ex Taq^®^ (Takara, Japan). The PCR products were subcloned into pDONR207 using the Gateway BP Clonase II enzyme mix (Thermo Fisher).

Sequenced genes were translated into protein sequences, aligned, and phylogenetic trees were built using Molecular Evolutionary Genetics Analysis version 7.0 (Kumar et al., 2016) with maximum likelihood methods and 1,000 bootstrap replicates.

All primers used are shown in **Supplementary Table S1**.

### Statistics

All data are presented as mean ± SD. Paired or unpaired two-tailed Student’s *t*-tests were used to compare group differences. *p*-values < 0.05 were considered significant. Three biological repeats were used for all analyses.

## Results

### Jujube saponins show different accumulation patterns in the seeds of different jujube varieties

**Figure 1:**
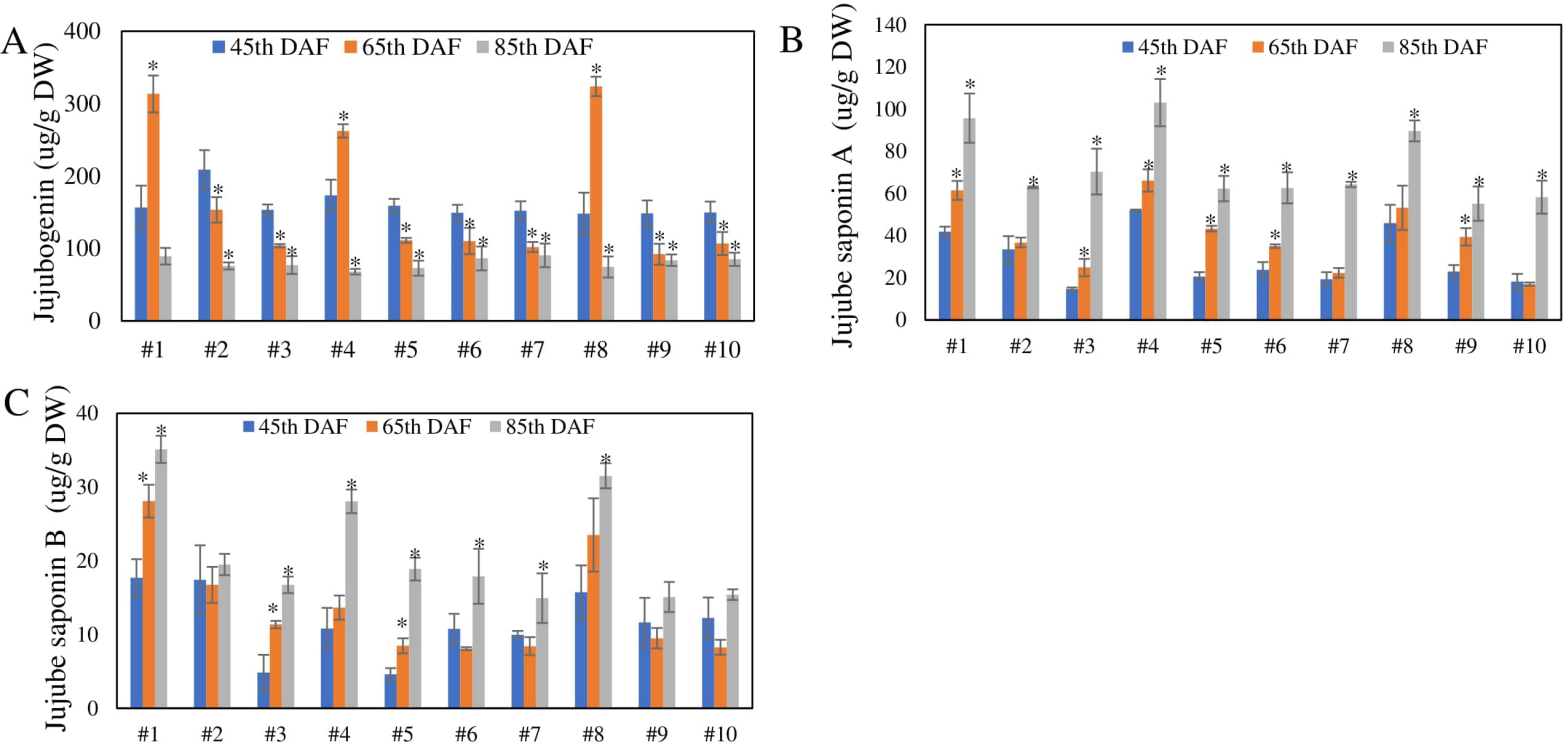
**(A)** Content of jujubogenin, **(B)** jujube saponin **(A, C)** jujube saponin B in 10 different varieties at the 45th, 65th, and 85th DAF. ^*^p < 0.05—significant level (*n* = 3).

The accumulation pattern of jujube saponins in seeds of different jujube varieties may vary. To verify this, we selected 10 different jujube varieties and detected the contents of jujube saponin A, jujube saponin B, and jujubogenin in seeds at 45, 65, and 85 DAF, respectively. The results showed that in seven of the 10 varieties, the content of jujubogenin decreased, while the content of jujube saponin A and B increased over time (**Figure 1**). However, varieties No. 1, No. 4, and No. 8 showed a different accumulation pattern. In these three varieties, accumulation of jujube saponins increased abnormally at 65 DAF compared with 45 DAF (**Figure 1**), and the content of jujube saponin A and B was relatively higher than in the other varieties during the red ripening stage These results indicate that the regulation of accumulation of jujube saponin A, jujube saponin B, and jujubogenin in the seeds of No. 1, No. 4, and No. 8 was different.

### Transcriptome and metabolome analyses reveal a significant difference

**Figure 2:**
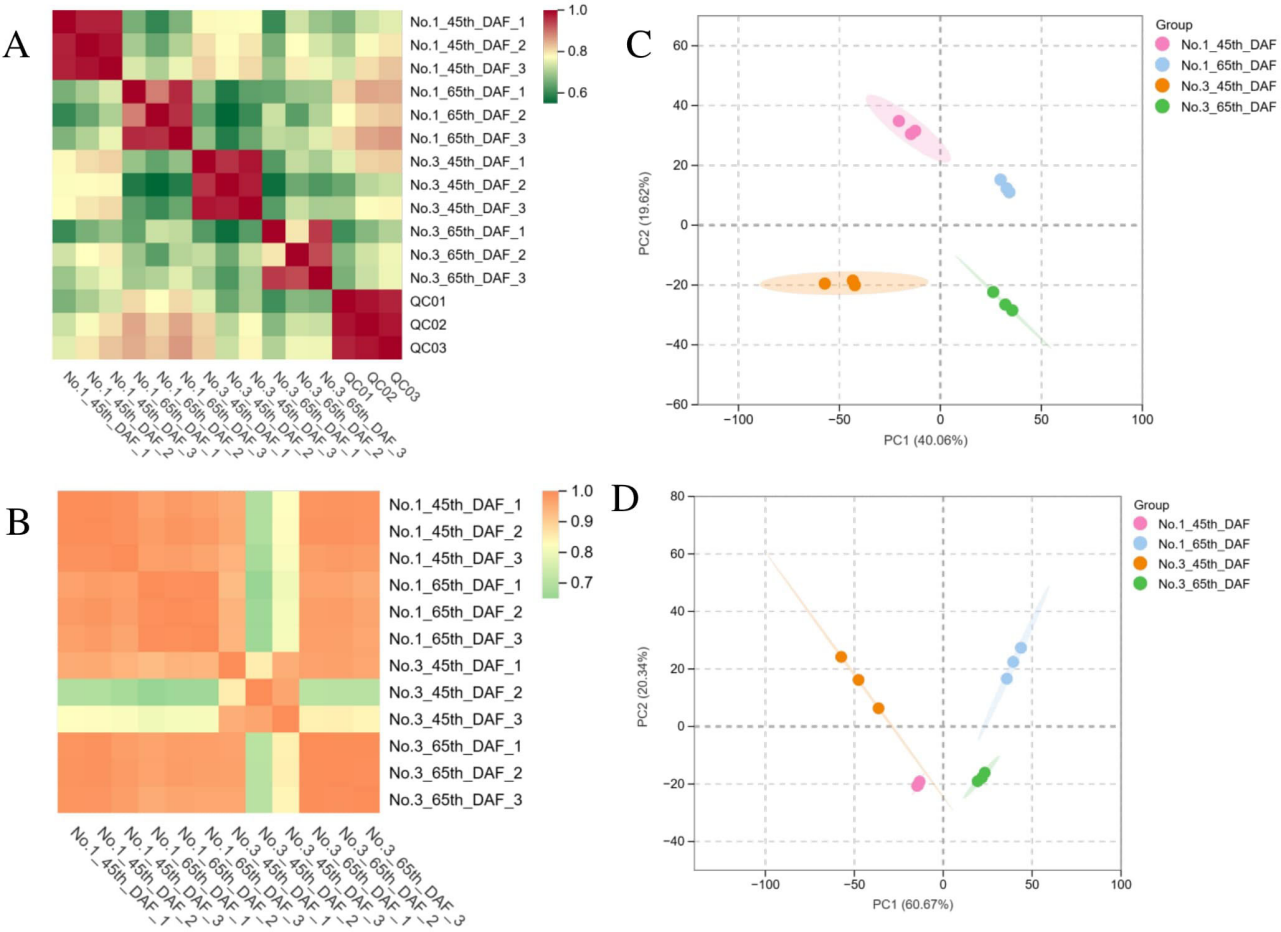
**(A)** Pearson correlation analysis of metabolome results. **(B)** Pearson correlation analysis of transcriptome results. **(C)** Principal component analysis of metabolome results. **(D)** Principal component analysis of transcriptome results.

To determine differentially expressed genes related to the accumulation of jujube saponins, we performed combined transcription–metabolome analysis in the seed of No. 1 and No. 3 jujube varieties at 45 and 65 DAF, which have significantly different contents of jujubogenin at 65 DAF. At the whole metabolome level, the difference caused by DAF is the main source of variation, while the difference caused by varieties is less pronounced, since PC1(40.6%) separated the 45 and 65 DAF groups, and PC2 (19.62%) separated the No. 1 and No. 3 groups (**Figure 2A**). Similar patterns were observed in PCA results at the whole transcriptome level (**Figure 2B**) and in the correlation matrix of metabolome and transcriptome analysis (**Figures 2C, D**).

**Figure 3:**
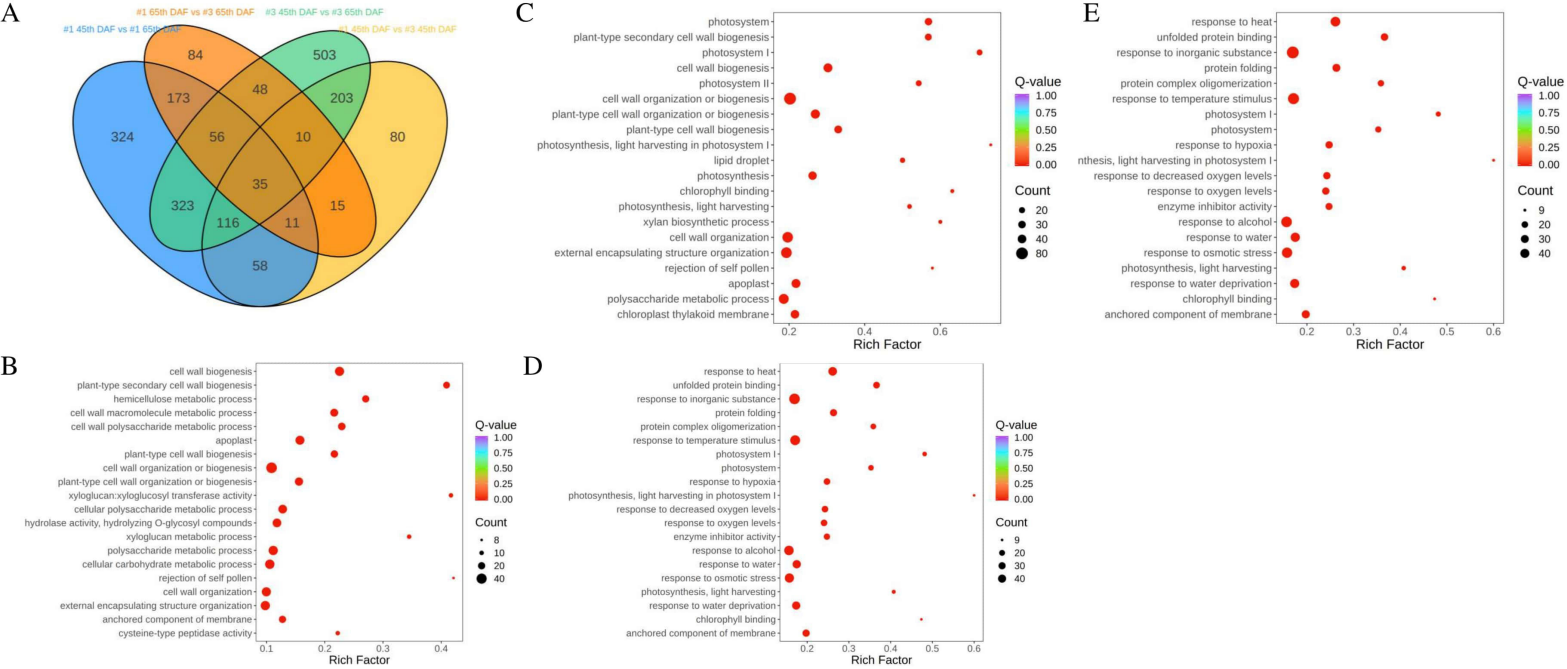
**(A)** Venn diagram of DEGs from all groups. GO analysis results. **(B)** No. 1 45th DAF vs. No. 1 65th DAF groups; **(C)** No. 3 45th DAF vs. No. 3 65th DAF groups. **(D)** No. 1 45th DAF vs. No. 3 45th DAF groups. **(E)** No. 1 65th DAF vs. No. 3 65th DAF groups.

**Figure 4:**
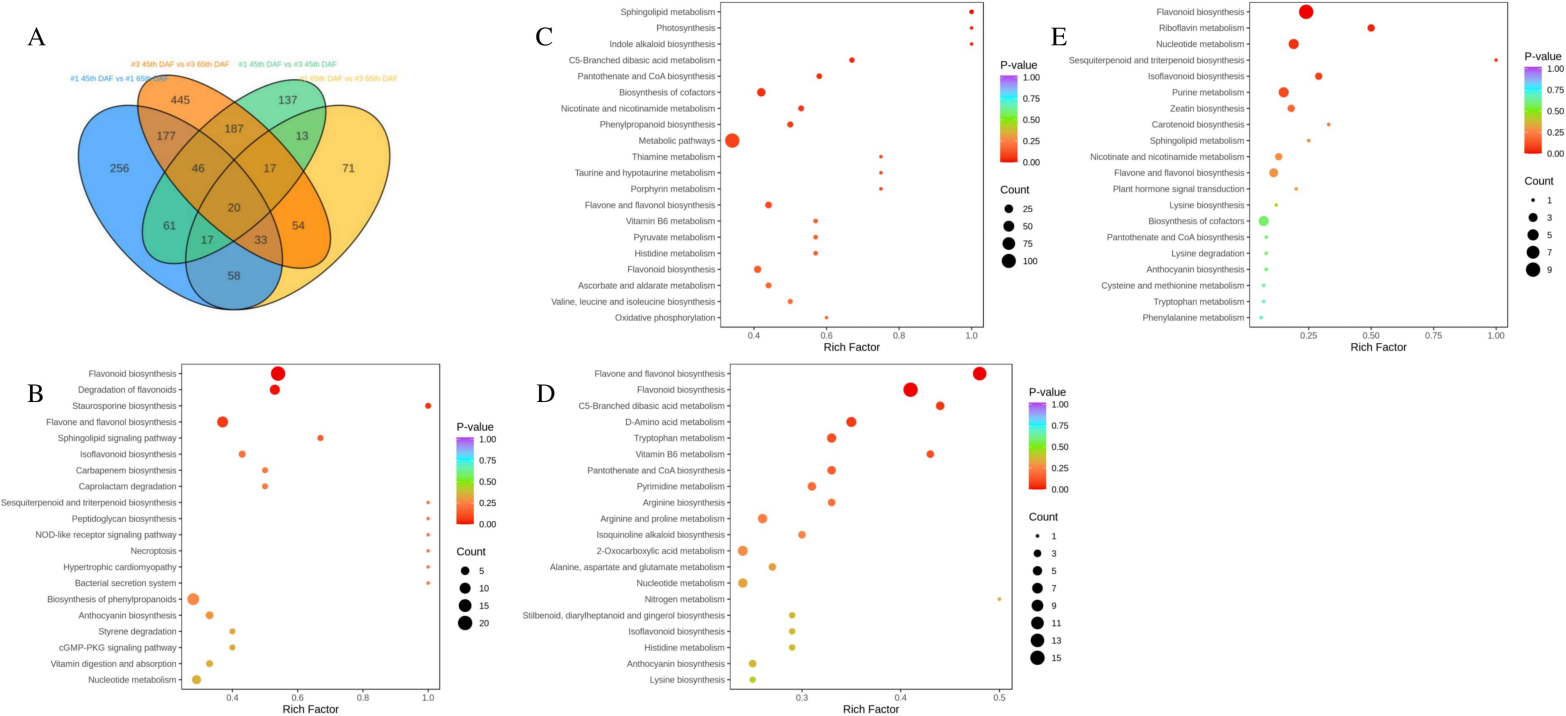
**(A)** Venn diagram of DAMs from all groups. KEGG analysis results of **(B)** No. 1 45th DAF vs. No. 1 65th DAF groups, **(C)** No. 3 45th DAF vs. No. 3 65th DAF groups, **(D)** No. 1 45th DAF vs. No. 3 45th DAF groups, and **(E)** No. 1 65th DAF vs. No. 3 65th DAF groups.

However, the content of jujube saponins in No. 1 and No. 3 varieties was significantly different at 45 and 65 DAF. Thus, we proceeded to determine which genes and metabolites were differentially expressed by varieties and DAF. Results showed 2,040 DEGs and 1,593 DAMs (**Supplementary Figures S1**, **S2**). Of all these DEGs and DAMs, 668 DAMs and 1,097 DEGs were discovered in No. 1–45 DAF vs. No. 1–65 DAF groups; 498 DAMs and 529 DEGs were discovered in No. 1–45 DAF vs. No. 3–45 DAF groups; 979 DAMs and 1,295 DEGs were discovered in No. 3–45 DAF vs. No. 3–65 DAF groups; and 283 DAMs and 433 DEGs were discovered in No. 1–65 DAF vs. No. 3–65 DAF groups (**Figures 3A**, **4A**). The datasets of metabolome and transcriptome results are shown in **Supplementary Tables S2**–**S6**, respectively.

### GO and KEGG analyses revealed significant differences between varieties in seeds

To discover the function of significant DEGs, GO and Kyoto Encyclopedia of Genes and Genomes (KEGG) analyses were performed on all DEGs. GO analysis results showed differences between varieties. DEGs in the No. 1–45 DAF vs. No. 1–65 DAF groups and No. 3–45 DAF vs. No. 3–65 DAF groups were annotated as related to cell wall biogenesis, photosystem, lipid droplet, and cysteine-type peptidase activity (**Figures 3C, D**). DEGs in the No. 1–45 DAF vs. No. 3–45 DAF groups and No. 1–65 DAF vs. No. 3–65 groups were annotated differently. In No. 1–45 DAF vs. No. 3–45 DAF groups, DEGs were annotated as associated with protein folding, photosystem, and response to abiotic stress (**Figure 3E**). In the No. 1–65 DAF vs. No. 3–65 DAF groups, DEGs were annotated as associated with protein folding, photosystem, response to abiotic stress, and enzyme inhibitor activity (**Figure 3E**).

KEGG analysis revealed significant differences between varieties. DEGs in the No. 1–45 DAF vs. No. 1–65 DAF comparison were annotated as associated with flavonoid biosynthesis, triterpenoid biosynthesis, and nucleotide metabolism (**Figure 4B**). DEGs in the No. 3–45 DAF vs. No. 3–65 DAF comparison were annotated as associated with flavonoid biosynthesis, vitamin biosynthesis, amino acid biosynthesis, taurine metabolism, and porphyrin metabolites (**Figure 4C**). By contrast, DEGs in the No. 1–45 DAF vs. No. 3–45 DAF and No. 1–65 DAF vs. No. 3–65 DAF exhibited similar trends: KEGG annotated these DEGs as associated with flavonoid biosynthesis, vitamin biosynthesis, amino acid biosynthesis, and triterpenoid biosynthesis (**Figures 4D, E**). These results indicate that the differential accumulation of jujube saponins was the result of differential expression of genes related to triterpenoid biosynthesis.

**Figure 5:**
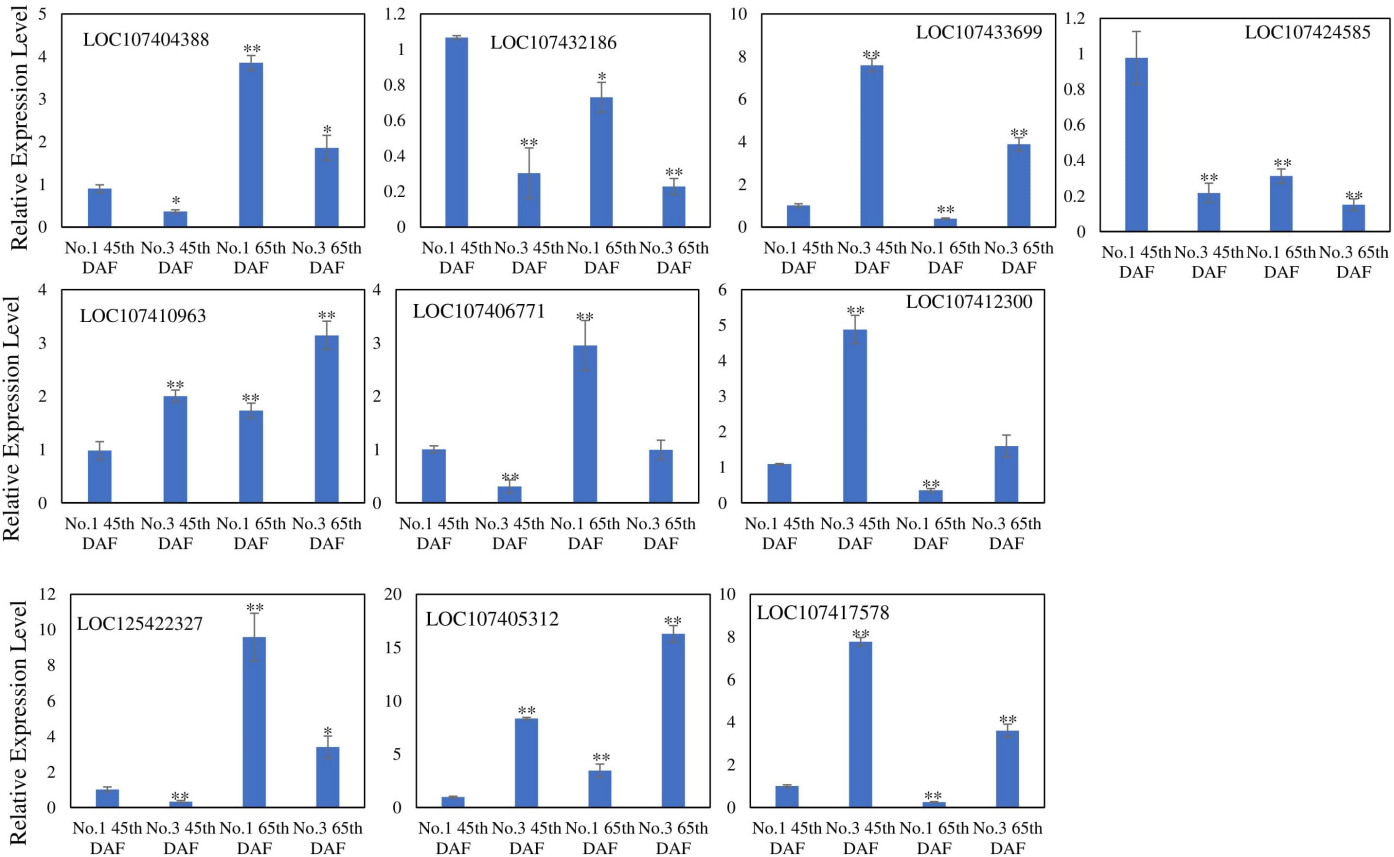
q-RT PCR analysis to confirm transcriptome results. ^*^p < 0.05; ^**^p < 0.01—significant levels (*n* = 3).

To confirm the DEG results, we randomly selected 10 DEGs with high FPKM and significantly higher relative expression levels for qRT-PCR analysis (**Figure 5**). These results are consistent with our transcriptome data.

### Potential genes related to jujube saponin biosynthesis discovered by GO analysis

**Figure 6:**
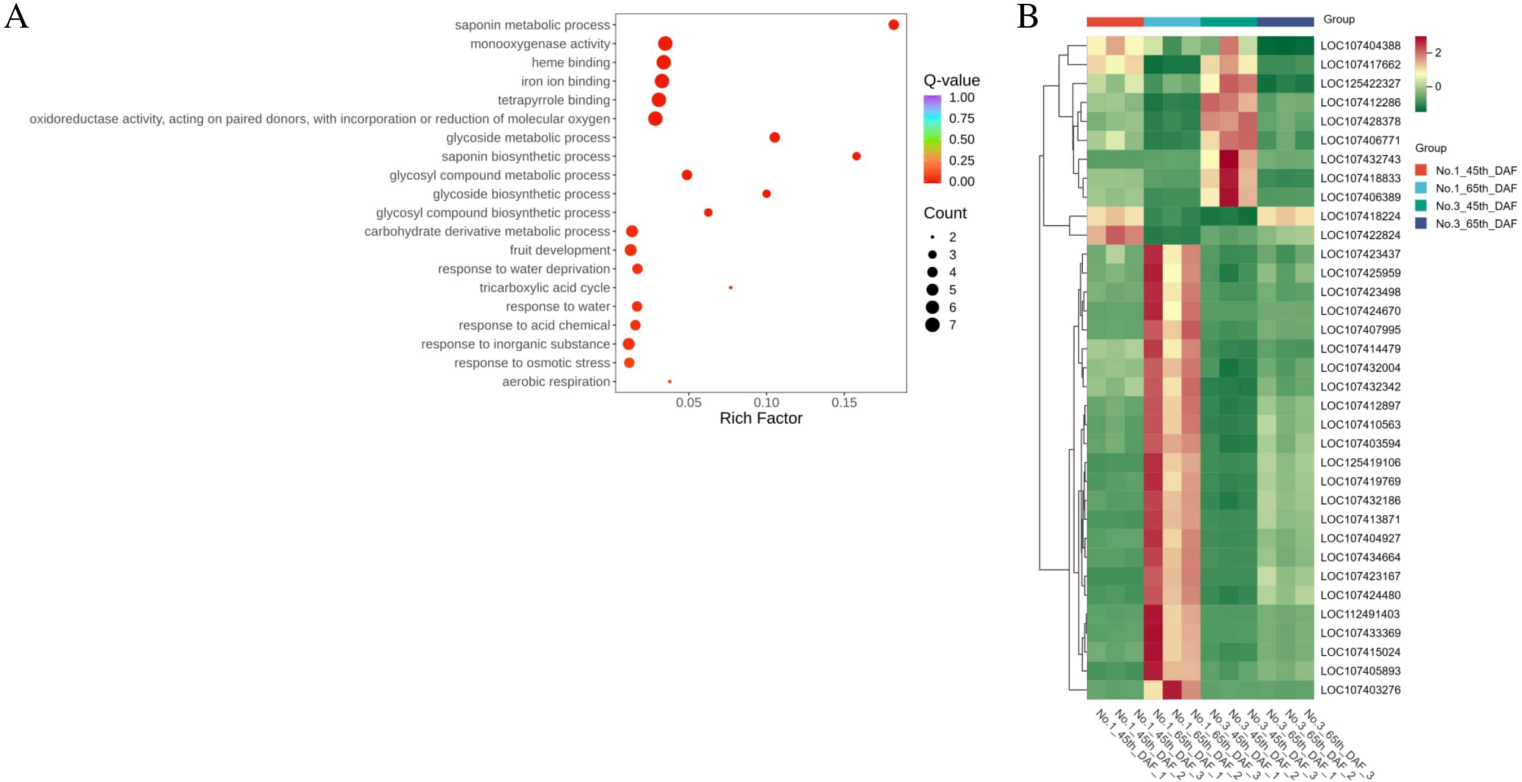
**(A)** GO analysis results and **(B)** heatmap of filtered genes.

Among all DAMs, 20 DAMs were significant in all groups, including jujube saponin A, jujube saponin B, and jujubogenin, which were located in the center of the DAMs Venn diagram (**Figure 4A**). Thus, 35 DEGs significant in all groups might contribute to the different accumulation of jujube saponin A, jujube saponin B, and jujubogenin between varieties and time points (**Figure 3A**). To functionally analyze these 35 DEGs, GO analysis was performed. The results showed that the DEGs were enriched in saponin metabolic process, monooxygenase activity, heme binding, iron ion binding, and glycoside metabolic process (**Figure 6A**). The heatmap showed that nine of these genes were upregulated in variety No. 3 compared with variety No. 1 and downregulated over time; two of these genes were upregulated in variety No. 3 compared with variety No. 1 and upregulated over time; and 24 genes were downregulated in variety No. 3 compared with variety No. 1 and upregulated over time (**Figure 6B**). These 35 genes were highly likely related to the biosynthesis of jujube saponin.

### Three genes involved in the saponin metabolic process were induced at 45 DAF and 65 DAF, but suppressed at 85 DAF

As dammaran-type triterpenes, jujube saponins share a synthetic pathway with ginsenosides. The main synthetic strategy that has not yet been elucidated is the conversion of dammarenediol-II to jujube saponins, which mainly involves the oxygenation and glycosylation of dammarenediol-II. These two-step reactions are usually catalyzed by enzymes of the CYP450 and UGT gene families, so we hypothesize that the CYP450 gene family catalyzes the oxidation of dammarenediol-II to jujubogenin.

To discover genes that catalyze the biosynthesis of jujubogenin, the expression level of three genes involved in the saponin metabolic process, LOC107404388, LOC125422327, and LOC107406771, were analyzed by qRT-PCR (**Figure 5**). Among these three genes, LOC107404388 was annotated as a UDP-glucosyltransferase 29-like gene, and LOC125422327 and LOC107406771 were annotated as CYP450 family genes. The expression levels of these three genes showed similar trends compared with 45 DAF in both varieties: increased at 65 DAF but suppressed at 85 DAF. Additionally, expression of these three genes was higher in variety No. 1 than in No. 3. These results indicate that these three genes might positively contribute to the biosynthesis of jujube saponins.

**Figure 7:**
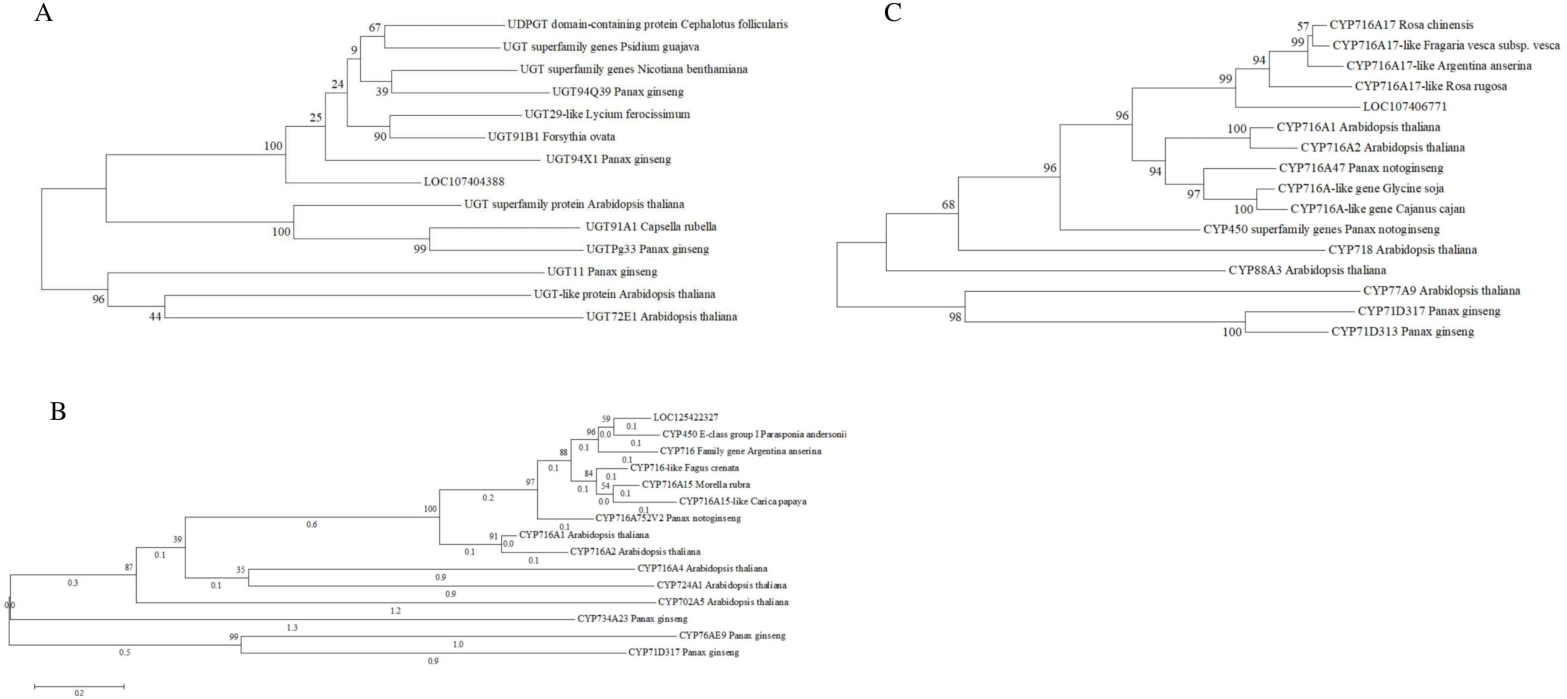
Phylogenetic trees of **(A)** LOC107404388, **(B)** LOC125422327, and **(C)** LOC107406771. Maximum likelihood (ML) was used to construct this tree with 1,000 replicate bootstrap support.

Based on the annotation, the CDS of LOC107404388, LOC125422327, and LOC107406771 were cloned, and a maximum likelihood tree of these three genes was constructed to confirm their annotation (**Figure 7**). Results showed that LOC107404388 is a UGT superfamily gene, while LOC125422327 and LOC107406771 are CYP716 family genes.

**Figure 8:**
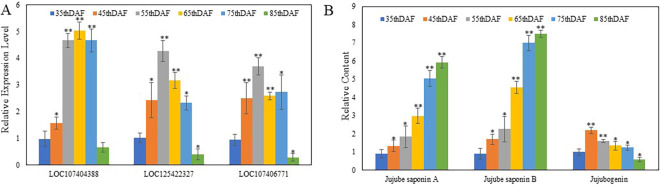
**(A)** Relative expression of LOC107404388, LOC125422327, and LOC107406771 and **(B)** relative content of jujube saponin **(A)** jujube saponin **(B)** and jujubogenin in variety No. 5. ^*^p < 0.05; ^**^p < 0.01—significant levels (*n* = 5).

Furthermore, the trend of expression levels of these three genes is consistent with the trend of jujube seed saponin content in variety No. 5 (**Figure 8**). From 35 to 85 DAF, expression of these three genes was significantly increased in 45, 55, 65, and 75 DAF compared with 35 DAF, then significantly decreased in 85 DAF. In contrast, LOC107404388 was only decreased at 85 DAF compared with 35 DAF. The relative contents of jujube saponin A, jujube saponin B, and jujubogenin showed similar trends: the content of jujubogenin was significantly increased at 45 DAF compared with 35 DAF, then decreased at 55, 65, 75, and 85 DAF compared with 45 DAF. The contents of jujube saponin A and jujube saponin B were significantly increased at 45, 55, 65, and 75 DAF compared with 35 DAF, then slightly increased at 85 DAF compared with 75 DAF. These results indicate that these three genes participate in the biosynthesis of jujube saponins.

## Discussion

### Stress response genes appear correlated with the biosynthesis of jujube saponins

Our data suggest that differences in the content of jujube saponins between varieties may be related to different stress tolerance. In No. 1–45 DAF vs. No. 3–45 DAF groups and No. 1–65 DAF vs. No. 3–65 DAF groups, genes related to response to abiotic stress were majorly downregulated in variety No. 3 compared with variety No. 1 (**Figure 3**), whereas the content of jujube saponins was higher in variety No. 1. These results indicate that abiotic stress tolerance appears to be correlated with the biosynthesis of jujube saponins.

Similar results were also reported in many studies on other Chinese medicinal plants. In *Panax ginseng*, stress-related genes ERDL6 were upregulated in older wild-simulated ginseng, which is rich in ginsenoside varieties and amounts (Kim et al., 2023). In *Scutellaria baicalensis*, drought and salt stress can induce the content of baicalin and baicalein (Su et al., 2018). Long-term stress induced expression of SbWRKY34, leading to a flavonoid index ingredient level in *Scutellaria baicalensis* that initially decreased, then rose as the drought duration extended, followed by a notable postrehydration increase in baicalin, wogonoside, and baicalein content and a decrease in levels of wogonin and oroxylin A (Zhang et al., 2025). In *Glycyrrhiza glabra*, controlled drought stress upregulates the expression of key genes like squalene synthase, β-amyrin synthase, and cycloartenol synthase involved in the biosynthesis of triterpenoid saponins and directly enhances the production of glycyrrhizin (Nasrollahi et al., 2014). In *Eucommia ulmoides*, EuRBG10 was induced by salt and drought stress and was proposed to play a hub role in regulating the biosynthesis of alkaloids (Zuo et al., 2022).

### Potential UDP-glucosyltransferase and CYP450s related to jujube saponin biosynthesis

As triterpenoids, intermediate metabolites in the biosynthesis of jujube saponins include mevalonate, farnesyl pyrophosphate (FPP), squalene, and dammarenediol-II. Based on our results, three genes related to the biosynthesis of jujube saponin were discovered by transcriptome–metabolome combined analysis. LOC125422327 and LOC107406771 were annotated as CYP716A15-like genes, and LOC107404388 was annotated as a UDP-glucosyltransferase 29-like gene. CYP716 subfamily members are multifunctional oxidases in triterpenoid biosynthesis. In *Medicago truncatula*, expression of CYP716 subfamily genes was highly correlated with that of β-amyrin synthase, which catalyzes the biosynthesis of oleanolic acid (Fukushima et al., 2013). In grape, CYP716A15 and CYP716A17 are involved in triterpenoid biosynthesis (Fukushima et al., 2011). CYP716A enzymes, including CYP716A752V2 isolated from olive, sugar beet, coffee, and *P. notoginseng*, were characterized as multifunctional C-28 oxidases and are involved in triterpenoid biosynthesis (Suzuki et al., 2018). CYP716A47 responds to JA and induces biosynthesis of ginsenoside in *P. ginseng* (Han et al., 2011) and *P. notoginseng* (Li et al., 2017). UDP-glucosyltransferase 29 (UGT29) is involved in the biosynthesis of triterpenoid saponin. In *P. ginseng*, UGTPg29 and its homolog Pq3-*O*-UGT2 can transfer glucose onto the C-2′ hydroxyl group of the first glucose residue at C-3 of ginsenoside Rh2 to produce ginsenoside Rg3 (Jung et al., 2014; Wang et al., 2015; Lu et al., 2017; Yang et al., 2020). These findings suggest that LOC125422327, LOC107406771, and LOC107404388 might be involved in the biosynthesis of jujube saponins.

In summary, we performed integrated transcriptomic and metabolomic analyses of seeds from 10 *Z. jujuba* varieties at 45, 65, and 85 DAF. Our analysis revealed that three candidate genes, LOC107404388, LOC125422327, and LOC107406771, exhibited expression patterns positively correlated with jujube saponin accumulation. These genes were significantly upregulated from 45 to 65 DAF but suppressed by 85 DAF. qRT-PCR validation confirmed their higher expression in high-saponin varieties vs. low-saponin varieties. Metabolite profiling further demonstrated coordinated accumulation of mevalonate, squalene, dammarenediol-II, and saponins in high-expression genotypes. Notably, varieties exhibiting enhanced saponin production showed downregulation of abiotic stress–response genes. Taken together, these results identify key glycosyltransferase and cytochrome P450 genes potentially governing jujuboside biosynthesis in *Z. jujuba*.

## Data Availability

The datasets presented in this study can be found in online repositories. The names of the repository/repositories and accession number(s) can be found below: https://ngdc.cncb.ac.cn/search/specific?db=bioproject&q=PRJCA046538, PRJCA046538.

## Supplementary material

Supplementary Figure 1 Heatmap of DEGs from all groups.

Supplementary Figure 2 Heatmap of DAMs from all groups.

Supplementary Table 1 Primers used in this paper.

Supplementary Table 2 Data sets of metabolome results.

Supplementary Table 3 Data sets of Different expression genes in No. 1–45 DAF vs No. 1–65 DAF group.

Supplementary Table 4 Data sets of Different expression genes in No. 1–65 DAF vs No. 3–65 DAF group.

Supplementary Table 5 Data sets of Different expression genes in No. 1–45 DAF vs No. 3–45 DAF group

Supplementary Table 6 Data sets of Different expression genes in No. 3–45 DAF vs No. 3–65 DAF group.
