# Supplementary figures and images for: Integrated transcriptome and metabolome analyses reveal key genes regulating jujuboside biosynthesis in Ziziphus jujuba var. spinosa
Source: Front Plant Sci. 2026 Jan 8;16:1708851. doi: 10.3389/fpls.2025.1708851 (PMC12823797; doi:10.3389/fpls.2025.1708851)

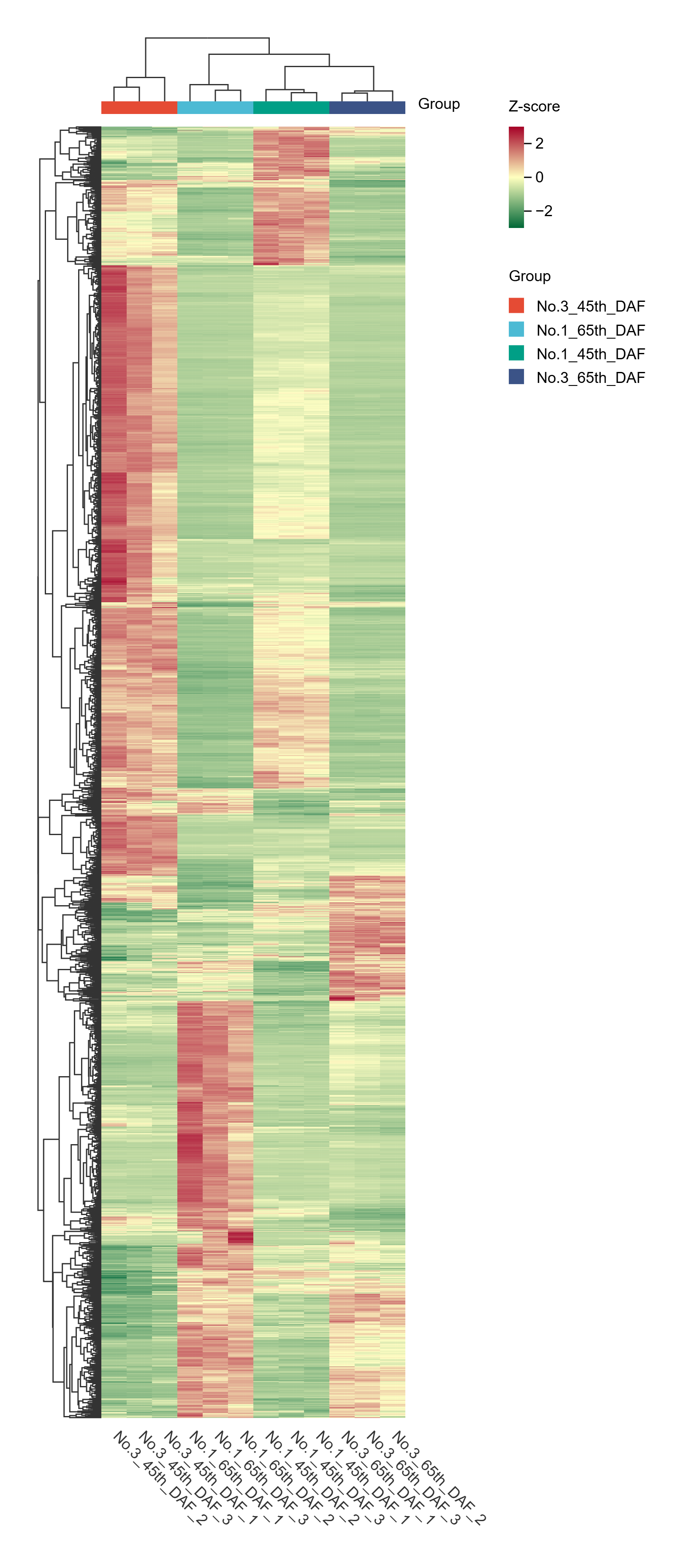

Supplement: Supplementary Figure 1 — Heatmap of DEGs from all groups. [file Image1.png]

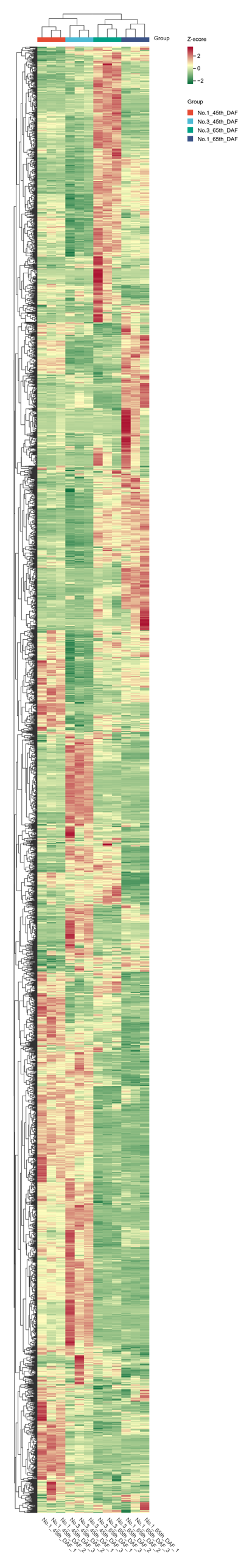

Supplement: Supplementary Figure 2 — Heatmap of DAMs from all groups. [file Image2.png]
